# The effect of *Malus doumeri* leaf flavonoids on oxidative stress injury induced by hydrogen peroxide (H_2_O_2_) in human embryonic kidney 293 T cells

**DOI:** 10.1186/s12906-020-03072-6

**Published:** 2020-09-11

**Authors:** Yanyan Li, Yunyi Li, Zhie Fang, Dan Huang, Yalin Yang, Dijia Zhao, Mingchun Hang, Junda Wang

**Affiliations:** 1 Pharmacy Department of Chongqing Traditional Chinese Medicine Hospital, Chongqing, 400021 People’s Republic of China; 2 Radiology Department of Chongqing Traditional Chinese Medicine Hospital, Chongqing, 400021 People’s Republic of China

**Keywords:** *Malus doumeri*, Flavonoid, Human embryonic kidney 293 T cells, mRNA, Oxidative stress

## Abstract

**Background:**

In this study, *Malus doumeri* leaf flavonoids (MDLF) were used as the research object to observe their in vitro antioxidant stress ability. Hydrogen peroxide (H_2_O_2_) was used to induce oxidative stress in 293 T cells.

**Methods:**

MTT, flow cytometry, and qPCR were used to verify the effect of MDLF.

**Results:**

In vitro cell experiments showed that at a concentration of 0–160 μg/mL, MDLF did not affect the normal proliferation of human embryonic kidney 293 T cells (HEK 293 T cells), and MDLF had no cytotoxic effect in this concentration range. It was found that MDLF could maintain the survival of HEK 293 T cells (82.6%) at a high concentration (160 μg/mL). Morphological observation also found that MDLF can inhibit the cell structure imperfection caused by H_2_O_2_. It was also observed that MDLF could significantly increase the levels of catalase (CAT), superoxide dismutase (SOD), glutathione (GSH), and glutathione peroxidase (GSH-Px) and reduce the level of malondialdehyde (MDA). The results of quantitative polymerase chain reaction (qPCR) showed that MDLF could significantly up-regulate the mRNA expression levels of CAT, SOD, GSH, GSH-Px, B-cell lymphoma-2 (Bcl-2) and downregulate the expression levels of B-cell lymphoma-2 associated x protein (Bax), tumor necrosis factor-alpha (TNF-α), and nuclear factor kappa-B (NF-κB) in oxidative stress-injured cells. The HPLC analysis showed that MDLF contained hyperin, isoquercetin, quercitrin, hesperidin, myricetin, baicalin and quercetin.

**Conclusion:**

From the experimental results, it was observed that MDLF has a strong anti-oxidation ability in vitro, and it can interfere with the oxidative stress damage caused by H_2_O_2_ in 293 T cells. Therefore, MDLF is a type of natural substance with good anti-oxidant effect, and it has the potential to interfere with many diseases.

## Background

The production of oxidative stress is due to the excessive production of free radicals and the release of a large number of reactive oxygen species (ROS) and other oxidizing substances after the body is injured by endogenous or exogenous factors. However, the ability of the body to resist oxidation is decreased and the accumulation of ROS cannot be inhibited, which leads to a imbalance between the ability of oxidation and anti-oxidation. The oxidation reaction of the body is far beyond the ability of antioxidant clearance. Excessive ROS caused by oxidative stress can stimulate cells to initiate lipid peroxidation reaction, which can damage cell function and structure [1, 2]. The concentration of intracellular Ca^2+^ is increased under oxidative stress. It increases the expression of the apoptotic gene, but it decreases the expression of the anti-apoptotic protein, leading to normal cell apoptosis and pathological changes in the body [3]. When infectious inflammation occurs, pathogens invade the body, activate neutrophils, increase oxygen consumption, and generate a large number of oxygen free radicals. At the same time, when pathogens invade and cause a stress reaction, they can promote the release of catecholamines and produce a large number of oxygen free radicals. The resulting oxidative stress reaction can lead to inflammatory infiltration of neutrophils and can produce a large number of pro-inflammatory mediators. In addition, cell death caused by oxidative stress can also activate the inflammatory cells [4]. Oxidative stress and inflammatory reaction can cause a variety of diseases, such as hypertension and atherosclerosis [5].

The body balance is disrupted after oxidative stress, and this causes many diseases. Under the stimulation by external adverse factors, the level of skin internal oxidation rises, resulting in the metabolic imbalance of the oxidation system, which directly or indirectly leads to the occurrence of some skin diseases, such as skin photoaging, skin tumors, pigmented diseases, and wound healing and repair [6]. Studies have shown that the involvement of oxidative stress in the pathogenesis of hypertension is mainly due to vascular damage. ROS accumulation caused by oxidative stress can lead to dysfunction of the vascular endothelium, dysfunction of vasoconstriction and relaxation, failure to regulate blood circulation, and increase in blood pressure [7]. Clinical studies have found that the more serious the incidence of type 2 diabetes, the lower the expression of glyoxal reductase 1 gene and blocked metabolism of fatty acids in patients, resulting in excessive ROS to promote the body’s oxidative stress response. Oxidative stress can disrupt the function of islet β cells and promote insulin resistance, which eventually leads to the occurrence of type 2 diabetes [8]. The accumulation of a large number of ROS caused by oxidative stress can affect the central nervous system. Oxidation of the central nervous system can lead to the occurrence of multicentric vascular diseases, such as atherosclerosis and heart failure [9]. Under oxidative stress, excessive ROS can activate hepatic stellate cells, increase the accumulation of extracellular matrix, damage the liver, further produce a large number of ROS, and aggravate liver fibrosis by regulating the signal transduction pathway [10]. Clinical research also noted that the oxidation level in cancer patients was higher than that in normal people [11]. Therefore, it is very important to regulate the level of oxidation and inhibit abnormal oxidative stress.

At present, many plants have been found to have antioxidant effects, which mainly arise from flavonoids, saponins, polysaccharides, and organic acids [12, 13]. Antioxidant plants restore the balance of the oxidation antioxidant system by scavenging excessive free radicals [14]. Most of the plants rich in flavonoids have a certain antioxidant effect, and most of the flavonoids have a certain antioxidant effect. The 2,3-double bond, 4-carbonyl, and 3 or 5-hydroxy in the structure of flavonoids make an important contribution to the antioxidant effect of flavonoids [15]. Flavonoids contained in plants can not only clear the free radicals in the chain initiation stage, but also directly capture the free radicals in the reaction chain, block the free radical chain reaction, play a dual role of prevention and chain breakage, and exert an antioxidant effect [16]. *Malus doumeri* is a *Rosaceae* family plant, whose fruit is consumed. The leaves are also used to a certain extent. Firstly, they are processed into drinks such as tea; secondly, they are used as folk drugs to prevent diarrhea; thirdly, they have antiseptic and bacteriostatic effects, and they are often mixed with other foods and drugs as natural preservatives [17]. Studies have shown that there are many chemical components in the leaves of *Malus* that are beneficial to human health; among them, the vitamin C content reaches 177.75 mg/g [18]; in addition, there are 12 free amino acids in the healthy tea made from Malus; among them, the methionine content is the highest [19]. The content of polyphenols in the leaves of *Malus doumeri* is about 5%, and the content of flavonoids in general tea is only 1–2% of the total polyphenols, while that in the leaves of *Malus doumeri* is more than 60%. After rough processing, the yield of total flavonoids in the dry leaves of *Malus doumeri* can be more than 3%, which shows that the content of flavonoids in the leaves of *Malus doumeri* is high [20].

Because of the limited area of application and cognition, there are few researches on *Malus doumeri*. However, with an increasing number of drinks and health products processed from the leaves of *Malus doumeri*, its biological activity and mechanism need to be studied further. In this study, the components of flavonoids in the leaves of *Malus doumeri* leaf (MDLF) were analyzed, and the effect of MDLF on oxidative stress injury induced by hydrogen peroxide (H_2_O_2_) in human embryonic kidney 293 T cells was studied for the first time, which is the theoretical basis for further utilization of MDLF accumulation.

## Methods

### Extraction of MDLF

After the dried *Malus doumeri* leaves (identification number: SC11442282300212, Enshi Xihaitang Biotechnology Co., Ltd., Enshi, Hubei, China) were crushed and sieved. A voucher specimen is deposited at the herbarium of the Chongqing University of Education under the number CCICFF-201902. Professor Xin Zhao authenticated the sample. Then 200 g of these leaves were weighed and placed in a beaker precisely and 70% ethanol was added according to the liquid to material ratio of 20:1; then they were placed in a 60 °C water bath for 3 h and the liquid was extracted for standby, passing through FL-3 macroporous resin. The liquid passing through the resin was evaporated to dryness through a rotary evaporator, and the extracted MDLF was obtained [21].

### Cytotoxicity of MDLF in HEK 293 T cells

After being resuscitated, HEK 293 T cells (Cell Bank of the Chinese Academy of Science, Shanghai, China) were inoculated in the deme medium containing 10% fetal bovine serum and 1% penicillin streptomycin double antibody. Then the cells were cultured in a carbon dioxide incubator (37 °C, 5% carbon dioxide, MCO-18 AC, Panasonic, Osaka, Japan) for 48 h and digested with 0.25% trypsin. Further, 293 T cells were counted and made into a 1 × 10^4^ cells/mL suspension; then 200 μL cell suspension was inoculated into a 96 well cell culture plate and cultured at 37 °C for 24 h until cells were attached to the wall. Then the culture medium was discarded, and 200 μL new des medium containing 0–200 μg/mL MDLF was added for 48 h. Then the medium in the 96 well culture plate was discarded, and the deme medium containing 5 mg/mL MTT (200 μL, Solarbio Life Sciences, Beijing, China) was added for continuous culture for 4 h. The medium was discarded again, dimethyl sulfoxide (DMSO, Solarbio Life Sciences) of 200 μL was added, and the reaction was carried out for 30 min without light and vibration. Finally, the optical density (OD) value of each hole was measured at 494 nm (Evolution™ 350, Thermo Fisher Scientific, New York, USA), and the cell survival rate (cell survival rate/% = (Ar) after the MDLF treatment was calculated / As) × 100%, where Ar: OD value of MDLF treated cells; As: OD value of normal cells) to observe the toxic effect of MDLF in 293 T cells [22].

### Effect of MDLF on H_2_O_2_-induced oxidative damage in HEK 293 T cells

Cells were resuscitated and cultured according to the experimental operation in the cytotoxicity experiment of MDLF on HEK 293 T cells. Cells were made into a cell suspension and cultured in 96 well plates. Cells were attached to the wall and then discarded. Further, 200 μL of deme medium containing 0.3 mmol/L H_2_O_2_ was added and cultured for 4 h to induce a cell oxidative damage model. After that, the medium was discarded again, the deme medium with concentration of 0–200 μg/mL MDLF was added to culture for 48 h, and then the OD value of each cell was measured at 494 nm according to the cytotoxicity experiment of MDLF on HEK 293 T cells and the inhibition effect of MDLF on oxidative damage induced by H_2_O_2_ in HEK 293 T cells was observed by calculating the survival rate of cells after oxidative damage prevention by each concentration of MDLF [22].

### Morphological observation of cells

According to the effect of MDLF on the oxidative damage of HEK 293 T cells induced by hydrogen peroxide, the cells were cultured, induced oxidative damage and treated with MDLF for 48 h. The morphology of cells in each group was observed under the microscope (EVOS M7000, Thermo Fisher Scientific, New York, USA).

### Observation of apoptosis by flow cytometry

According to the experimental operation of MDLF on oxidative damage induced by H_2_O_2_ in HEK 293 T cells, the cells were cultured and treated with MDLF for 48 h; then the cells were fixed, and apoptosis was detected by flow cytometry after staining (AccuriC6, BD Biosciences, San Jose, CA, USA) [23].

### Determination of MDA, SOD, GSH, GSH PX, and CAT levels in HEK 293 T cells

According to the experimental operation of MDLF on oxidative damage induced by H_2_O_2_ in HEK 293 T cells, the cells were cultured and treated with MDLF for 48 h. Then the MDA, SOD, GSH, GSH-Px, and CAT levels in cells of each group were measured according to the kit instructions (Nanjing Jiancheng Bioengineering Institute, Nanjing City, China) [24].

### qPCR method

In the experiment of oxidative damage induced by H_2_O_2_ in HEK 293 T cells by MDLF, cells in each group were used to extract total RNA of cells by Trizol reagent (Solarbio Life Sciences). Further, 1 μL of oligo (DT) 18 primer (500 ng/μL, Solarbio Life Sciences) and 1.0 μL of total RNA (1.0 μg/μL) were added to 10.0 μL of nuclease free water and heated in PCR for 5 min (65 °C). Then, 4.0 μL of 5 × reaction buffer, 1.0 μL of ribolock RNase inhibitor (20 U), 2.0 μL of 10 mM dNTP mix, and 1.0 μL of reversible reverse transcription (200 U/μL) were added to the above reaction system solution for cDNA transcription (60 min at 42 °C, 5 min at 70 °C). Then, 1 μL 10.0 μM upstream primer and 1 μL 10.0 μM downstream primer (Table 1, Thermo Fisher Scientific) and 7.0 μL sterile double distilled water were added to the 1.0 μL reaction fluid for amplification. The reaction conditions were 95 °C denaturation for 3 min, 60 °C annealing for 30 s, and 95 °C extension for 1 s. The relative expression intensity of each gene to be tested and the control cell (hydrogen peroxide induced oxidative damage cell) was calculated by the 2^-∆∆Ct^ method through the measured CT value [24].

**Table 1:** The primer seque quantitative polymerase chain reaction

Gene name	Sequence
SOD	Forward: 5′-AGATGGTGTGGCCGATGTGT-3′ Reverse: 5′-TCCAGCGTTTCCTGTCTTTGTA-3′
CAT	Forward: 5′-TGTTGCTGGAGAATCGGGTTC-3′ Reverse: 5′-TCCCAGTTACCATCTTCTGTGTA-3′
GSH	Forward: 5′-TACGGCTCACCCAATGCTC-3′ Reverse: 5′-CTATGGCACGCTGGTCAAATA-3′
GSH-Px	Forward: 5′-GTCGGTGTATGCCTTCTCGG-3′ Reverse: 5′-CTGCAGCTCGTTCATCTGGG-3′
Bax	Forward: 5′-AAGCTGAGCGAGTGTCTCCGGCG-3′ Reverse: 5′-CAGATGCCGGTTCAGGTACTC AGTC-3′
Bcl-2	Forward: 5′-ATGTGTGTGGAGAGCGTCAACC-3′ Reverse: 5′-CAGAGACAGCCAGGAGAAATCAA-3′
TNF-α	Forward: 5′-CACGCTCTTCTGCCTGCT-3′ Reverse: 5′-GCTTGTCACTCGGGGTTC-3′
NF-κB	Forward: 5′-GAAGCACGAATGACAGAGGC-3′ Reverse: 5′-GCTTGGCGGATTAGCTCTTTT-3′
GAPDH	Forward: 5′- TCA AGA AGG TGG TGA AGC AGG-3′ Reverse: 5′- AGC GTC AAA GGT GGA GGA GTG-3′

### Main components of MDLF in high-performance liquid chromatography (HPLC)

MDLF was dissolved in DMSO to prepare a sample solution with a concentration of 10 mg/ml; then it was diluted with 50% methanol to prepare a solution to be tested with a concentration of 2 mg/mL and the solution to be tested was filtered by a 0.22 μm organic filter membrane and tested on the machine. The detection conditions were chromatographic column: Thermo Scientific Accucore C18 (4.6 mm × 150 mm, 2.6 μm), gradient elution mobile phase C was acetonitrile; mobile phase B was 0.5% glacial acetic acid aqueous solution; flow rate was 0.5 mL/min, column temperature was 35 °C; detection wavelength was 360 nm; and injection volume was 10 μL. Then the content of MDLF was calculated by the external standard peak area method according to the HPLC method (UltiMate3000 HPLC System, Thermo Fisher Scientific), and the formula was as follows: ${M}_x={C}_{r\times}\frac{A_x}{A_r}\times C$ M_C_=C_1_ × A_1_ × C÷A_R_.

Where: Mc is the content of flavonoids in the sample, mg/g; C1 is the concentration of the standard, mg/ml; Ar is the peak area of the standard; A1 is the peak area of the sample; C is the concentration of the original solution of the sample, 0.0025 g/mL.

### Data processing

All experiments were carried out three times in parallel. The mean ± standard deviation (SD) was used to express the results. SAS9.1 statistical software was used to analyze the significant differences between groups of data at the level of *P* < 0.05. The analysis method was Duncan’s multiple range test single factor analysis of variance (ANOVA).

## Results

### Toxic effect of MDLF in HEK 293 T cells

**Fig. 1:**
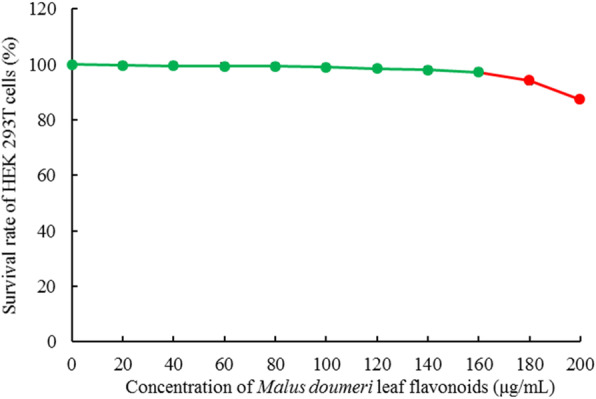
Toxic effect of *Malus doumeri* leaf flavonoids on human embryonic kidney 293 T cells. Values presented are the mean ± standard deviation

When HEK 293 T cells were treated with MDLF at concentrations of 20, 40, 60, 80, 100, 120, 140, 160, 180, and 200 μg/mL, compared with HEK 293 T cells that were not treated with MDLF, MDLF at concentrations of 20–160 μg/mL did not significantly affect the proliferation of HEK 293 T cells, and the survival rate of HEK 293 T cells in this concentration range was more than 95% (Fig. 1). Therefore, 40, 80, and 160 μg/mL MDLF were used as sample concentrations for further tests.

### Effect of MDLF on oxidative stress injury induced by H_2_O_2_ in HEK 293 T cells

**Fig. 2:**
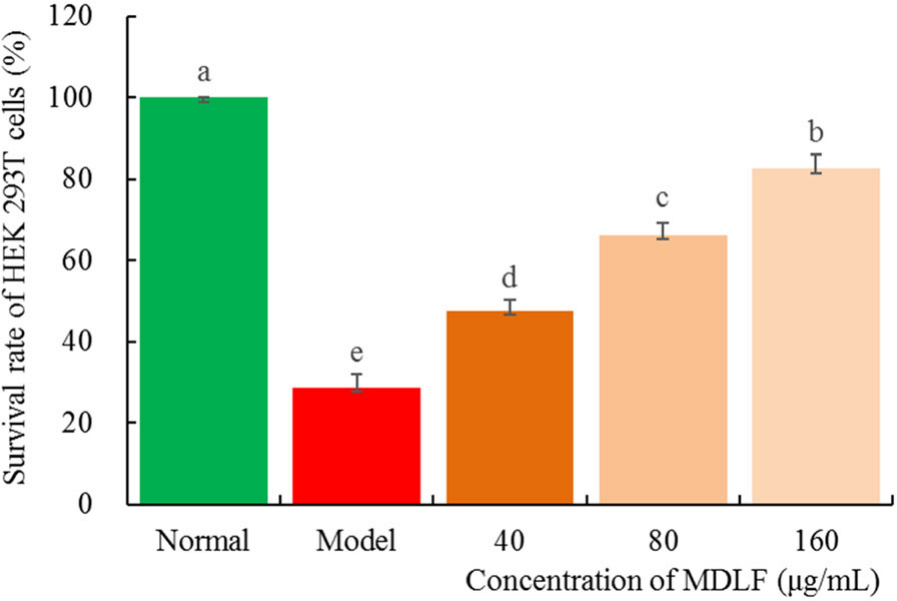
Effect of *Malus doumeri* leaf flavonoids on hydrogen peroxide induced oxidative damage of human embryonic kidney 293 T cells. Values presented are the mean ± standard deviation. Different letters indicate significant differences from each other at the level of *p* < 0.05 according to Tukey’s honestly significant difference. Normal: untreated human embryonic kidney 293 T cells; model: hydrogen peroxide induced oxidative damage human embryonic kidney 293 T cells

The survival rate of HEK 293 T cells treated with H_2_O_2_ was significantly lower than that of HEK 293 T cells treated without H_2_O_2_, and the survival rate was only 23.7% (Fig. 2). The survival rate of HEK 293 T cells increased with an increase in the concentration of MDLF. When the concentration of MDLF was 160 μg/mL, the survival rate of HEK 293 T cells increased to 82.6%.

### Effect of MDLF on the change in morphology induced by H_2_O_2_ in HEK 293 T cells

**Fig. 3:**
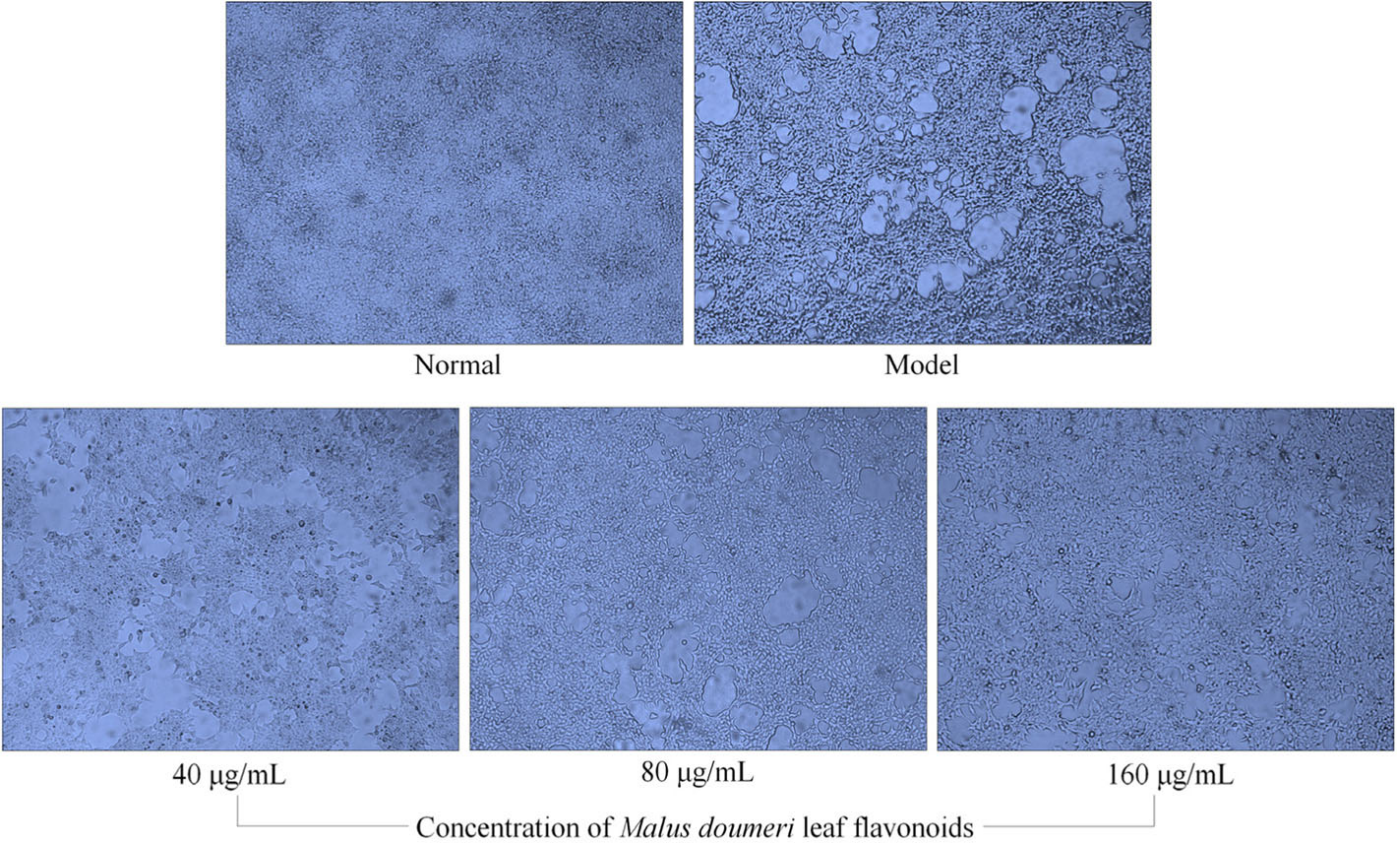
Morphological observation of hydrogen peroxide damaged human embryonic kidney 293 T cells. Normal: untreated human embryonic kidney 293 T cells; model: hydrogen peroxide induced oxidative damage human embryonic kidney 293 T cells

After cell culture, the normal HEK 293 T cells were found to be abundant in quantity, complete in cell structure, abundant in cytoplasm and orderly in cell arrangement in a microscopic field (Fig. 3); after being treated with H_2_O_2_, the number of HEK 293 T cells was decreased compared with the number of cells without H_2_O_2_ treatment. The cell morphology was severely atrophied, the cell structure was changed, and the cell damage was severe. MDLF could obviously improve the abnormal cell structure caused by H_2_O_2_ and could restore the cell structure and quantity. With an increase in MDLF concentration, the recovery of cells was better.

### Effect of MDLF on apoptosis induced by H_2_O_2_ in HEK 293 T cells

**Fig. 4:**
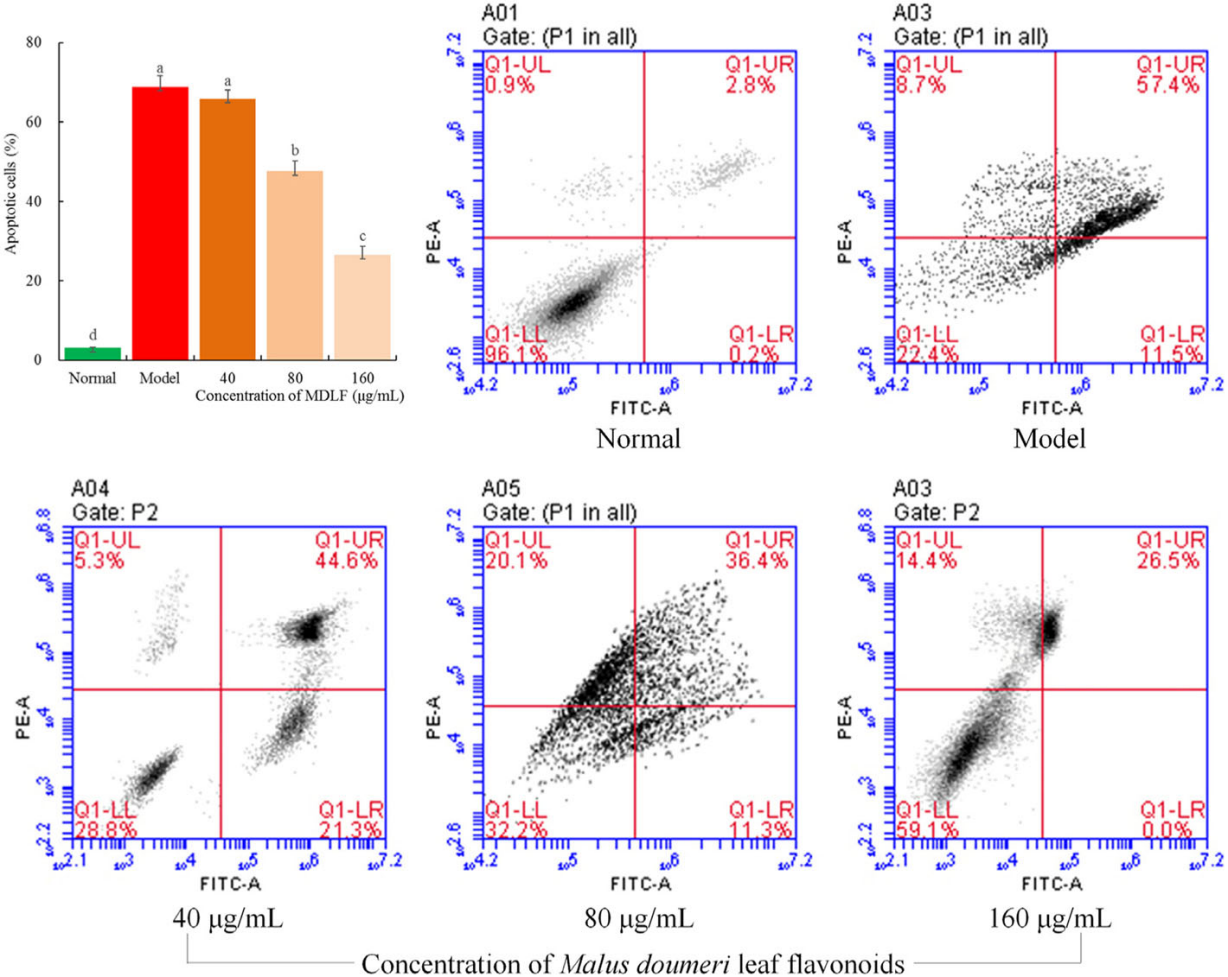
Observation of apoptosis on hydrogen peroxide damaged human embryonic kidney 293 T cells by flow cytometry. Values presented are the mean ± standard deviation. Different letters indicate significant differences from each other at the level of *p* < 0.05 according to Tukey’s honestly significant difference. Normal: untreated human embryonic kidney 293 T cells; model: hydrogen peroxide induced oxidative damage human embryonic kidney 293 T cells

HEK 293 T cells exposed to H_2_O_2_ showed a high level of apoptosis compared with those not exposed to H_2_O_2_ (Fig. 4). After oxidative stress injury, HEK 293 T cells exposed to MDLF at concentrations of 40, 80, and 160 μg/mL showed decreased apoptosis and improved growth of cells. The higher the concentration of MDLF, the better the cell status and the lesser the number of apoptotic cells.

### Effects of MDLF on the levels of MDA, SOD, GSH, GSH-Px, and CAT in HEK 293 T cells treated with H_2_O_2_

**Fig. 5:**
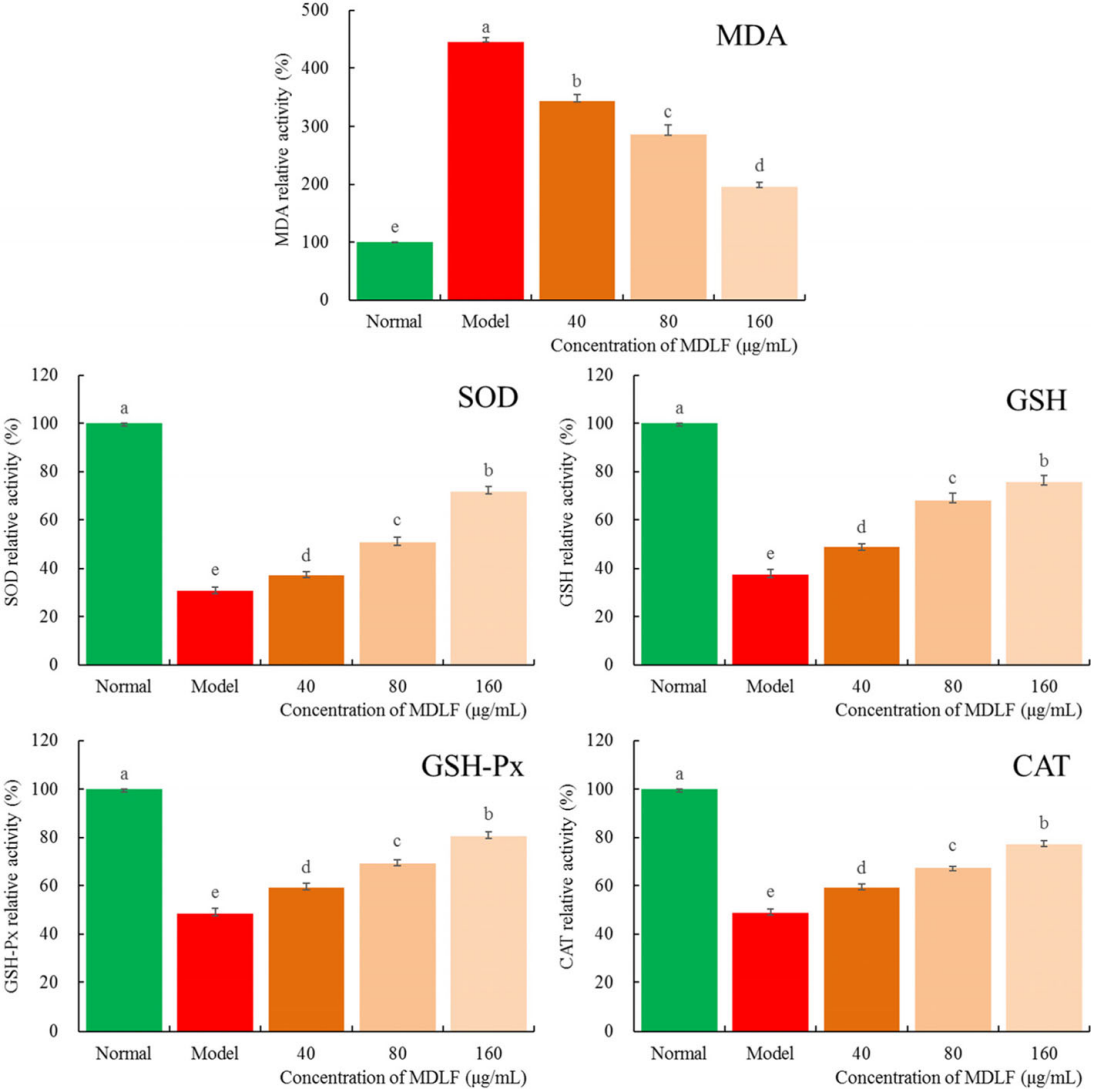
MDA, SOD, GSH, GSH-Px and CAT levels of hydrogen peroxide damaged human embryonic kidney 293 T cells. Values presented are the mean ± standard deviation. Different letters indicate significant differences from each other at the level of *p* < 0.05 according to Tukey’s honestly significant difference. Normal: untreated human embryonic kidney 293 T cells; model: hydrogen peroxide induced oxidative damage human embryonic kidney 293 T cells

The MDA level in HEK 293 T cells treated with H_2_O_2_ was significantly higher than that in cells not treated with H_2_O_2_ (*P* < 0.05), while the SOD, GSH, GSH-Px, and CAT levels were significantly lower (P < 0.05, Fig. 5). The concentration of MDLF at 40, 80, and 160 μg/ml decreased the MDA level and increased the levels of SOD, GSH, GSH PX, and CAT in the cells treated with H_2_O_2_. Among them, the concentration of MDLF at 160 μg/mL restored the MDA, SOD, GSH, GSH-Px, and CAT levels in the cells closest to those in normal HEK 293 T cells.

### Effect of MDLF on the expression levels of SOD, CAT, GSH, and GSH-Px mRNA induced by H_2_O_2_ in HEK 293 T cells

**Fig. 6:**
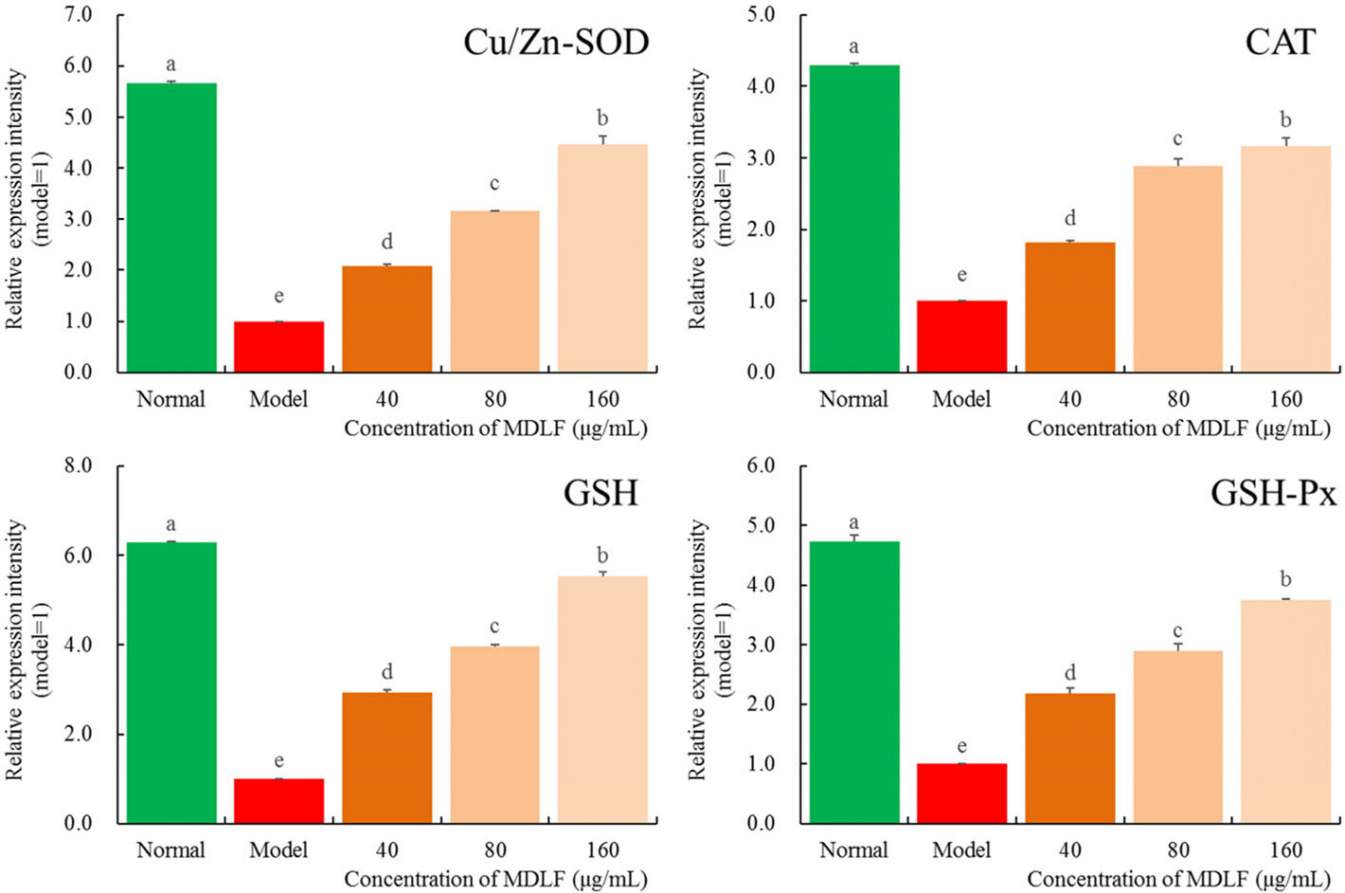
SOD, CAT, GSH and GSH-Px mRNA expression level of hydrogen peroxide damaged human embryonic kidney 293 T cells. Different letters indicate significant differences from each other at the level of *p* < 0.05 according to Tukey’s honestly significant difference. Normal: untreated human embryonic kidney 293 T cells; model: hydrogen peroxide induced oxidative damage human embryonic kidney 293 T cells

The levels of SOD, CAT, GSH, and GSH-Px mRNA expression in HEK 293 T cells treated with H_2_O_2_ were significantly lower than those in cells not treated with H_2_O_2_ (*P* < 0.05, Fig. 6). Different concentrations of MDLF could up-regulate the expression levels of SOD, CAT, GSH, and GSH-Px mRNA in H_2_O_2_ treated cells. With an increase in the concentration of MDLF, the expression levels of SOD, CAT, GSH, and GSH-Px in oxidatively damaged HEK 293 T cells were up-regulated. The results of the qPCR test were consistent with those of the kit test.

### Effect of MDLF on the expression levels of apoptosis-related Bax and Bcl-2 mRNA in HEK 293 T cells

**Fig. 7:**
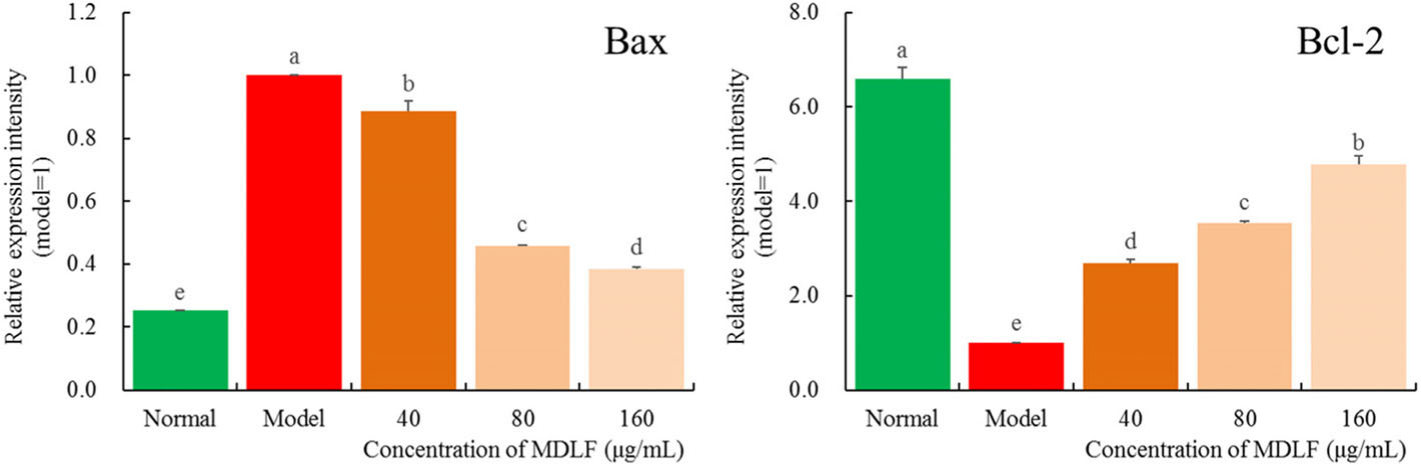
Bax and Bcl-2 mRNA expression level of hydrogen peroxide damaged human embryonic kidney 293 T cells. Different letters indicate significant differences from each other at the level of *p* < 0.05 according to Tukey’s honestly significant difference. Normal: untreated human embryonic kidney 293 T cells; model: hydrogen peroxide induced oxidative damage human embryonic kidney 293 T cells

The expression level of Bax mRNA in HEK 293 T cells was significantly increased (*P* < 0.05, Fig. 7) and the expression level of Bcl-2 was significantly decreased. MDLF could interfere with the abnormal expression of Bax and Bcl-2 mRNA caused by H_2_O_2_ in HEK 293 T cells; it up- regulated the expression of Bcl-2 and downregulated the expression of Bax.

### Effect of MDLF on the expression levels of TNF-α and NF-κB mRNA induced by H_2_O_2_ in HEK 293 T cells

**Fig. 8:**
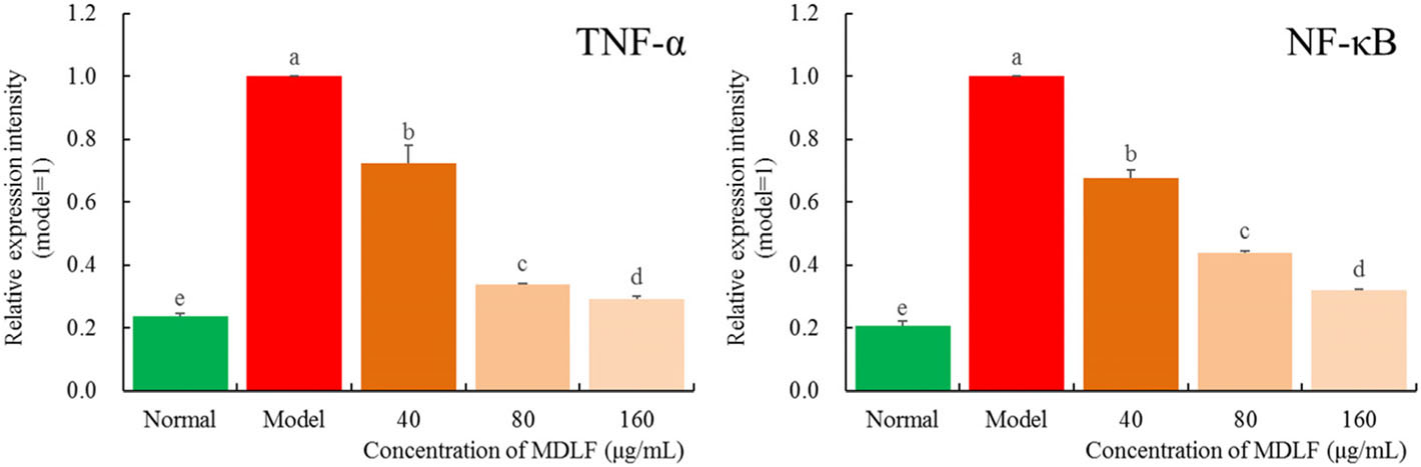
TNF-α and NF-κB mRNA expression level of hydrogen peroxide damaged human embryonic kidney 293 T cells. Different letters indicate significant differences from each other at the level of *p* < 0.05 according to Tukey’s honestly significant difference. Normal: untreated human embryonic kidney 293 T cells; model: hydrogen peroxide induced oxidative damage human embryonic kidney 293 T cells

The expression levels of TNF-α and NF-κB mRNA in HEK 293 T cells treated with H_2_O_2_ were up-regulated compared to those in cells treated without H_2_O_2_ (*P* < 0.05, Fig. 8). MDLF could significantly inhibit the expression of TNF-α and NF-κB induced by H_2_O_2_ in HEK 293 T cells. The higher the concentration of MDLF, the stronger the effect.

### Analysis of the main components of MDLF

**Fig. 9:**
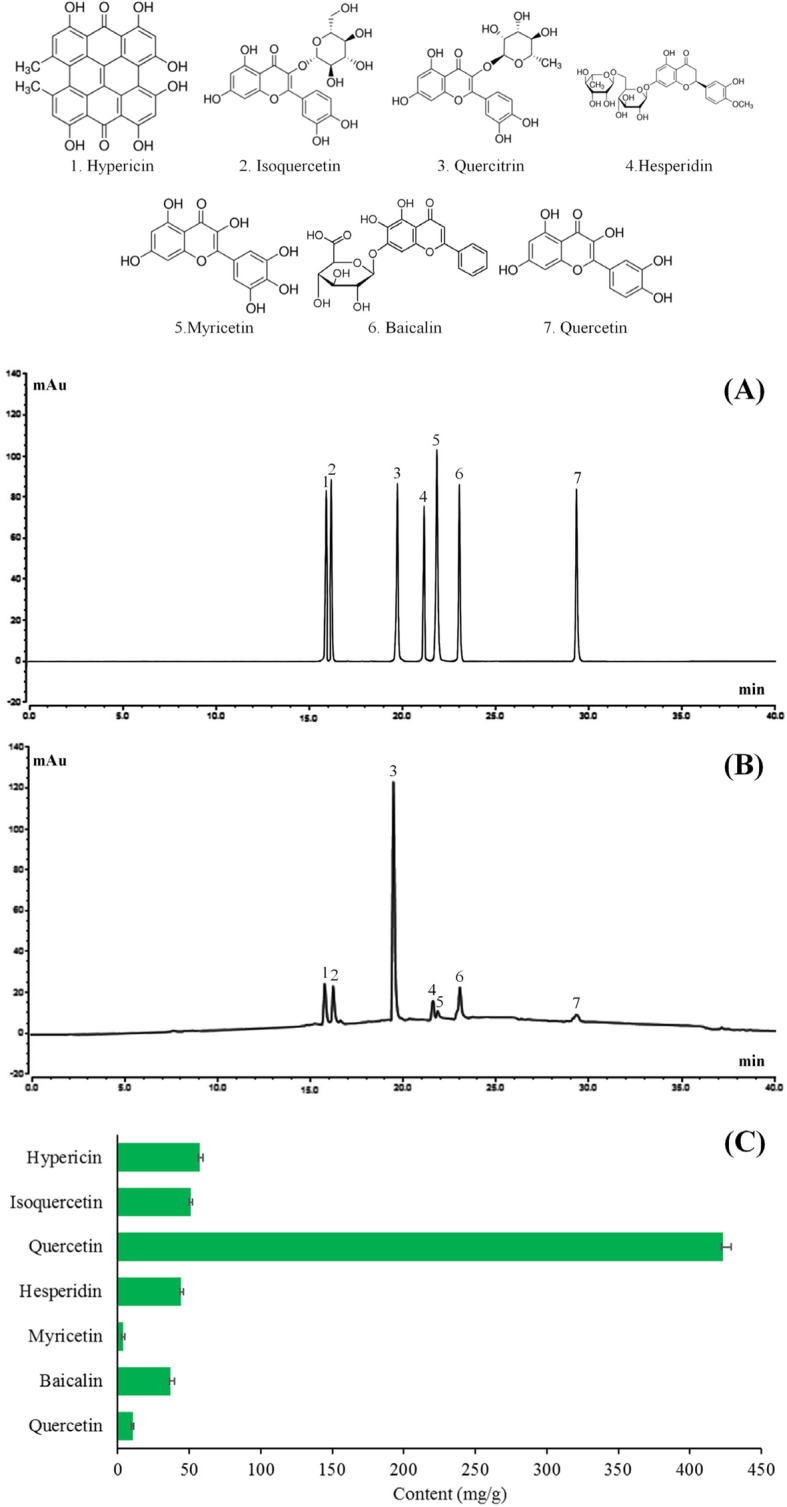
Composition analysis of *Malus doumeri* leaf flavonoids. (**a**) HPLC chromatogram of standards; (**b**) HPLC chromatogram of *Malus doumeri* leaf flavonoids; (**c**) content of components

HPLC analysis showed that MDLF mainly contained 7 compounds, namely hypericin, isoquercetin, quercitrin, hesperidin, myricetin, baicalin and quercetin (Fig. 9). Their contents in MDLF were 67.193, 58.063, 381.858, 12.447, 5.828, 20.135, and 17.391 mg/g, respectively.

## Discussion

As a traditional Chinese medicine or tea substitute, plant leaves have the characteristics of little or no side effects, and play an important role in human health. *Malus doumeri* Leaf is also a plant with traditional medicinal effects, but its mechanism of action has been less studied [18]. In this study, the antioxidant effect and mechanism of *Malus doumeri* leaf flavonoids in vitro were preliminarily studied.

After the occurrence of oxidative stress, normal metabolism and increment of tissue cells are affected. In serious cases, a large number of cells die, and proliferation slows down or even stagnates [25]. At the same time, abnormal oxidative stress leads to the destruction of normal cell structure, apoptosis of cells, and then abnormal cell death. Serious decline affects human health, and even threatens life. Reducing the degree of apoptosis caused by oxidative stress can protect the tissues and cells, and resist the attack of diseases on the body [26]. In this study, MDLF has been proved to inhibit abnormal cell death and apoptosis caused by oxidative stress in vitro; thus, it protect the cells.

MDA is recognized as a marker of oxidative stress. MDA released from the cell membrane can react with protein and nucleic acid, causing cross-linking polymerization and making normal synthesis of the protein impossible. MDA can also cause abnormal structure and function of the cell membrane, thus causing an abnormal physiological state of the body [27, 28]. SOD, GSH Px, and CAT are antioxidant enzymes in vivo. MDA is highly cytotoxic and can inhibit antioxidant enzymes in vivo. Oxidative stress caused by abnormal expression of these indicators is involved in the pathogenesis of many clinical diseases [29]. GSH is also an active peptide with good antioxidant effect, which can effectively regulate the oxidation balance of the body and inhibit the damage caused by oxidative stress [30]. The results of this study also showed that MDLF can interfere with the abnormality of oxidation-related indexes after H_2_O_2_ treatment of cells, and thus, it reduces the cell damage caused by oxidative stress and plays a role in protecting the normal cells.

Oxidative stress is closely related to cell apoptosis, which is mainly related to the mitochondrial pathway, the mitogen activated protein kinase (MAPK) pathway, endoplasmic reticulum stress, NF-κB, and Bcl-2 family [31]. In the Bcl-2 family, there are inhibitory factors, such as Bcl-2, and pro-apoptotic factors, such as Bax, and both of them have a synergistic effect. Bcl-2 is an antioxidant present in the body, which has an antioxidant effect and can protect the normal cells [32]. The results showed that the expression of Bcl-2 was increased significantly, while Bax expression was decreased under the action of antioxidants. Apoptosis of renal tubular epithelial cells after ureteral obstruction could be reduced by regulating Bax and bcl-2. This study also showed a similar effect. MDLF can intervene in the oxidative stress of HEK 293 T cells by enhancing the expression of Bcl-2 and reducing the expression of Bax.

Oxidative stress and inflammation promote each other. Research shows that TNF-α can enhance the expression of nicotinamide adenine dinucleotide phosphate (NADPH) oxidase and inhibit the expression of SOD, which leads to an oxidative stress reaction. However, inhibiting the expression of TNF-α can effectively inhibit the oxidative stress reaction [33]. NF-κB is also an important inflammatory factor. NF-κB plays a key role in many inflammatory reactions and immune responses. The incorrect regulation of NF-κB can lead to autoimmune diseases, chronic inflammation, and a variety of cancers [34]. In addition, oxidative stress can also activate the NF-κB signaling pathway. Activated NF-κB enters the nucleus and combines with c-myc and other apoptosis-related genes to promote gene transcription and cell apoptosis [35]. In this study, MDLF could also downregulate the inflammatory expression of HEK 293 T cells, so as to intervene in the relationship between oxidative stress and inflammation and protect the cells.

Hypericin, isoquercetin, quercitrin, hesperidin, myricetin, baicalin, and quercetin are commonly found flavonoids in plants, and all of them have strong antioxidant activity [36–42]. Hyperoside can exert many physiological activities, such as anti-inflammatory, anti-hypertensive, cholesterol lowering, and central pain relieving activities, and protective effects on the heart and brain vessels through its antioxidant capacity [36]. Isoquercetin and quercitrin have antioxidant, antitumor, hypoglycemic, and hypolipidemic effects, and they especially have significant inhibitory effects on inflammatory factors [37, 38]. Hesperidin has anti-inflammatory and antiviral effects, as well as certain antibacterial effects [39]. Myricetin has many biological activities, such as anti-inflammatory, anti-tumor, and anti-mutation activities; caries prevention; anti-oxidation; and elimination of free radicals in vivo [40]. Baicalin has antibacterial, anti-inflammatory, and cholesterol reducing effects, especially in liver disease and kidney disease [41]. Quercetin is not only used as an antioxidant in the food industry, but it also has anti-inflammatory and anti-cough effects [42]. MDLF contains these seven compounds. Their direct interaction constitutes the biological activity of MDLF, which confers a good ability to resist oxidative stress and the potential to prevent and treat other diseases. These flavonoids produce stable semiquinone free radicals through the reaction of phenolic hydroxyl group with free radicals, thus terminating the chain reaction of free radicals, which is the main mechanism for flavonoids to scavenge free radicals [43]. Phenolic hydroxyl group is the main active group of flavonoids. To a certain extent, the increase of phenolic hydroxyl group will increase the antioxidant activity; B-ring is the main active part of flavonoids to scavenge free radicals. When there is o-hydroxy group in the ring, the antioxidant activity is greatly improved; the 2,3-position double bond is beneficial to the formation of more stable radicals after the p-ring is de electron; 4-position carbonyl group can form hydrogen bond with o-hydroxyl, which makes free radical intermediate more stable; 3,5-hydroxyl belongs to synergic phenolic hydroxyl [44]. At the same time, flavonoids combine with cell membrane in the form of hydrogen bond to protect the unsaturated bond of cell membrane from contacting with free radicals, thus playing the role of antioxidant protection [45]. In this study, the MDLF may also play a role in avoiding human embryonic kidney 293 T cells damage by hydrogen peroxide.

## Conclusion

In this study, the results showed that MDLF could protect HEK 293 T cells from oxidative stress by regulating oxidation, apoptosis, and inflammation. MDLF contains active flavonoid substances, which make it play these roles. This study preliminarily verified the effect of MDLF, but its antioxidative effect and mechanism in vivo still need to be studied further. In the future, an in-depth study is needed to assess the role of MDLF in the intervention for various diseases through its antioxidant effect.

## Abbreviations

MDLF: *Malus doumeri* leaf flavonoids
H_2_O_2_: hydrogen peroxide
qPCR: quantitative polymerase chain reaction
MTT: 3-(4,5-dimethyl-2-thiazolyl)-2,5-diphenyl-2-H-tetrazolium bromide
HEK 293 T cells: human embryonic kidney 293 T cells
CAT: catalase
SOD: superoxide dismutase
GSH: glutathione
GSH-Px: glutathione peroxidase
MDA: malondialdehyde
Bcl-2: B-cell lymphoma-2
Bax: B-cell lymphoma-2 associated x protein
TNF-α: tumor necrosis factor-alpha
NF-κB: nuclear factor kappa-B
HPLC: high performance liquid chromatography
